# Research Progress of Methods for Degradation of Bisphenol A

**DOI:** 10.3390/molecules28248028

**Published:** 2023-12-09

**Authors:** Ying Han, Hao Dai, Xiaolong Rong, Haixia Jiang, Yingang Xue

**Affiliations:** School of Environmental Science and Engineering, Changzhou University, Changzhou 213164, China; huahuashijie0608@163.com (H.D.); forthefuture2021@126.com (X.R.); woshijiangxiaoye@gmail.com (H.J.)

**Keywords:** degradation of BPA, VOSviewer, adsorption

## Abstract

Bisphenol A (BPA), an endocrine disruptor widely used in industrial production, is found in various environmental sources. Despite numerous reports on BPA degradation and removal, the details remain unclear. This paper aims to address this gap by providing a comprehensive review of BPA degradation methods, focusing on biological, physical, and chemical treatments and the factors that affect the degradation of BPA. Firstly, the paper uses VOSviewer software (version 1.6.15) to map out the literature on BPA degradation published in the past 20 years, which reveals the trends and research focus in this field. Next, the advantages and limitations of different BPA degradation methods are discussed. Overall, this review highlights the importance of BPA degradation to protect the environment and human health. The paper provides significant insights for researchers and policymakers to develop better approaches for BPA degradation and removal.

## 1. Introduction

Bisphenol A (BPA), consisting of two phenol functional groups, is an endocrine-disrupting compound that is sparingly soluble in water but dissolves readily in organic solvents, impacting its behavior in the environment and its transport properties [1,2]. It is also widely used as an industrial chemical in the production of plastics and epoxy resins such as food containers, plastic water bottles, additives for thermos paper, adhesives, baby pacifiers, and dental fillers, and its applications and properties can vary based on its use in different materials [3,4,5]. BPA has potential adverse effects on endocrine disruption, developmental and reproductive effects, health concerns, and environmental impact [6,7]. Therefore, there has been a global consensus to prohibit the use of BPA, and countries such as the United States and Canada have enacted legislation to ban the sale of baby bottles containing BPA to prevent infants from being affected by BPA leakage [8].

BPA can degrade over time, especially when exposed to heat, UV light, or certain chemicals. This degradation can lead to the release of BPA from products into the environment. Given the potential adverse effects of BPA on human health and the environment, there is increasing emphasis on developing effective strategies to degrade this compound. The degradation of BPA involves breaking down its molecular structure into simpler and less harmful compounds. Different methods, including biological, chemical, and physical approaches, have been explored to degrade BPA and mitigate its harmful effects on the environment [9]. This paper primarily discusses the toxicity of BPA to humans and animals and its physical and chemical properties. It presents an overview of the latest research and advances in BPA degradation methods in three categories: biodegradation, physical adsorption, and chemical oxidation, including their effectiveness in degrading BPA and the challenges faced in implementing them. Ultimately, this review summarizes and infers future development trends in the degradation of BPA.

## 2. Properties and Characteristics of BPA

### 2.1. Physicochemical Properties of BPA

**Figure 1:**
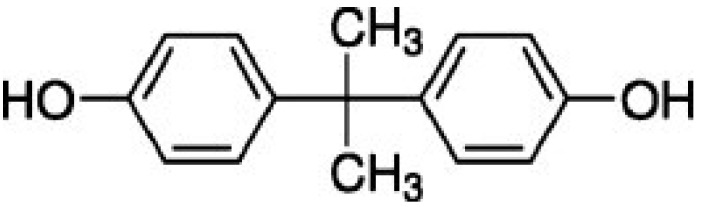
Chemical structure of BPA [10].

BPA, a derivative of phenol and acetone, is synthesized under acidic conditions with the aid of a catalyst. Its chemical nomenclature is 2,2-bis (4-hydroxyphenyl) propane. Found in its pure state as a white powder with a subtle odor, BPA exhibits solubility in various organic solvents such as ethanol, acetone, and benzene while showing near-insolubility in water. Noteworthy physical characteristics include a melting point range of 150~155 °C and a boiling point of 220 °C (0.53 kPa). Additionally, BPA is slightly soluble in carbon tetrachloride and undergoes decomposition at approximately 260 °C. Its moderate lipophilicity is reflected in a Log Kow value of 3.32, where Kow represents the n-octanol water partition coefficient. The molecular formula of BPA is C_15_H_16_O_2_, with a molecular weight of 228.29 g/mol. Figure 1 depicts the chemical structure, illustrating the connection of two hydroxyl groups to a central carbon atom, flanked by two phenyl groups.

### 2.2. Application and Harmful of BPA

BPA serves as a key constituent in numerous plastic materials, playing a pivotal role in the production of items like food, beverage containers, baby bottles, and cups [11,12]. Beyond its application in plastics, BPA is a crucial component in the manufacturing of various high-molecular-weight chemical materials. This includes its use in the production of epoxy resin, commonly employed for the internal coating of beverage cans, as well as polyester resin, polysulfone resin, polyphenylene ether resin, and unsaturated polyester resin. Additionally, BPA is used in the production of fine chemical products such as PVC stabilizers, plasticizers, flame retardants, plastic antioxidants, heat stabilizers, banana antioxidants, ultraviolet absorbers, agricultural fungicides, pesticides, and coatings in industrial operations [13].

**Figure 2:**
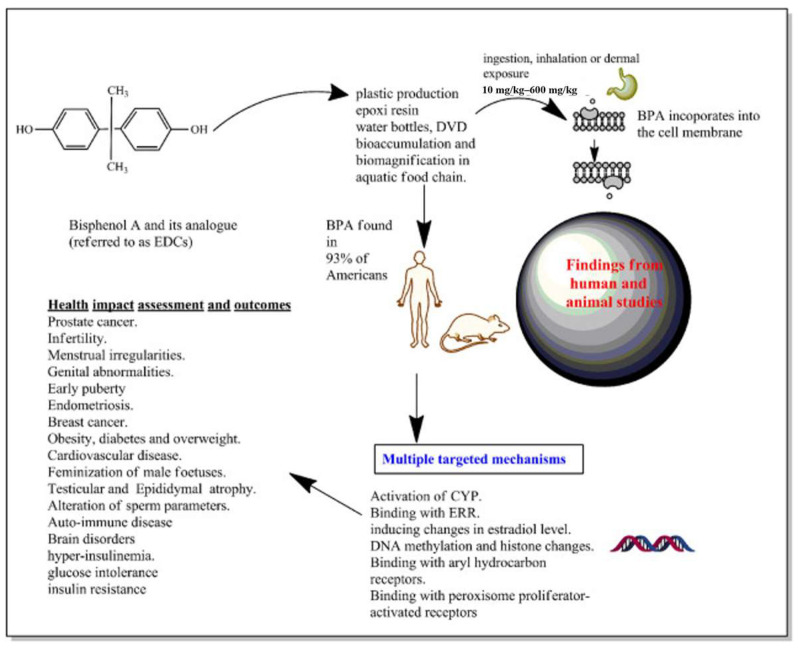
The endocrine activity mechanism of BPA and the health effects of exposure to BPA on humans and animals were introduced [20].

BPA can enter the body through the food chain and interact with the estrogen receptor (ER) to disrupt the production, release, transportation, metabolism, and other functions of normal hormones in the body, thereby affecting reproductive, neural, immune, and other bodily functions [14,15]. BPA can also block hormone signaling pathways by inhibiting the binding of hormones to their receptors, leading to a loss of function [16]. Current research indicates that exposure to BPA may lead to a decrease in male sperm count and an elevated incidence of endocrine-related disorders, such as early puberty in women, obesity, and cancer. Additionally, BPA exposure has been linked to an increased risk of premature birth and a higher rate of fetal malformations [17,18]. Figure 2 illustrates the endocrine effects of BPA and the potential health consequences of exposure to this substance in humans and animals. BPA is a compound that mimics estrogen and can bind to estrogen receptors in the body, thereby impacting the synthesis, transportation, and metabolism of natural estrogen. This disruption can lead to reproductive dysfunction, developmental abnormalities, immune and nervous system disorders, malignant tumors, and even cancer [19].

BPA not only leads to carcinogenesis but also exhibits estrogenic activity. Even in minute doses, it can induce premature puberty, reduce sperm count, and lead to prostate hyperplasia in women. Furthermore, BPA has been identified as embryotoxic and teratogenic in animals, significantly elevating the incidence of ovarian cancer, prostate cancer, leukemia, and other cancers in animals. In mice, BPA has been associated with asthma in offspring. Therefore, special attention should be paid to BPA intake during pregnancy, and regular monitoring of BPA levels in the body should be conducted, referencing recommended efflux levels post-intervention. Overall, the release of BPA into the environment poses a significant risk to both human and animal health [21,22,23].

## 3. Progress in the BPA Degradation

### 3.1. Analysis Results of VOSviewer

VOSviewer software (version 1.6.15) was utilized to collect articles focused on the degradation of BPA published over the past 20 years in the Web of Science (WOS) database.

**Figure 3:**
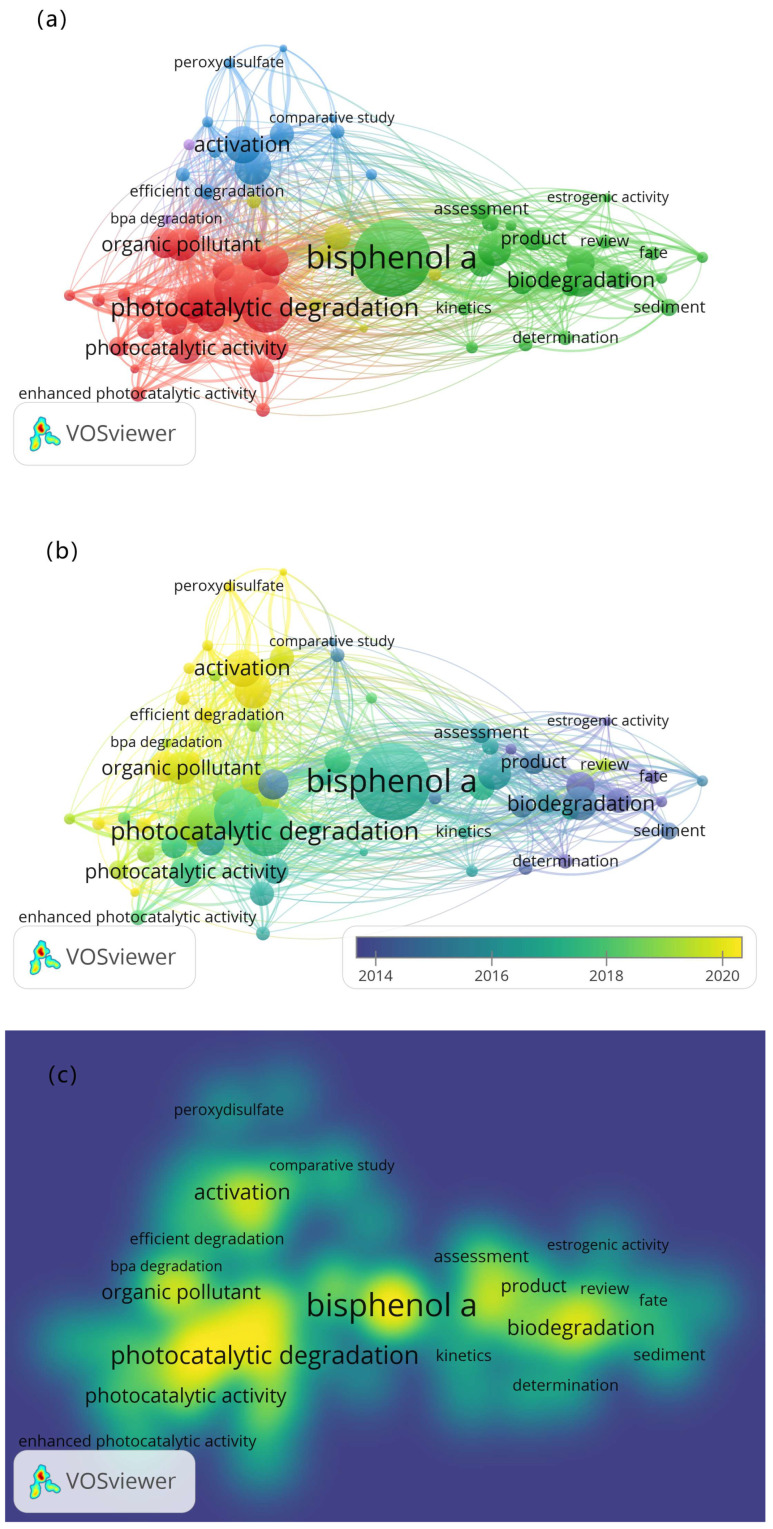
(**a**) Network (**b**) Overlay (**c**) Density visualization of degradation of BPA.

Figure 3a shows the network visualization diagram of BPA. To ensure accuracy and visibility, approximately 3000 articles were collected from WOS and uncommon keywords were eliminated. Figure 3a indicates that BPA is the most extensively studied bisphenol. Currently, the degradation of BPA is categorized into three main groups: biological degradation, physical adsorption, and chemical oxidation. Biodegradation involves the use of bacteria, laccase, and peroxidase to break down BPA. In physical adsorption, materials such as graphene, black phosphorus nanosheets, activated carbon, and membranes are employed for adsorption. Chemical oxidation includes advanced oxidation and photocatalysis. Advanced oxidation techniques primarily rely on the potent oxidation of hydroxyl radicals and sulfate radicals. Photocatalysis utilizes materials like titanium, zinc, silver, carbon, bismuth, and modified forms of these materials to construct composites that effectively degrade BPA.

Figure 3b illustrates the time-span diagram describing the development of BPA degradation in the three categories mentioned earlier. In recent years, laccase, peroxidase, nanoparticle-based materials such as graphene, and various photocatalysts have gained popularity for the degradation of BPA. Figure 3c reveals that the magnetic field for BPA degradation is the broadest, indicating why the degradation of BPA is the principal focus of this investigation.

### 3.2. Biological Treatment for BPA Degradation

In recent years, the use of biological systems for degrading refractory organic pollutants has emerged as an alternative to physical and chemical treatment methods [24,25]. While there are many biological methods for degradation, this paper focuses on BPA degradation using bacteria, laccase, and peroxidase. Compared to other BPA degradation methods, bacteria-assisted BPA degradation has a more significant environmental benefit. By harnessing microorganisms naturally present in the external environment, the cost of BPA degradation can be reduced. Biological systems can produce a degradation effect through their enzymes [26]. Enzyme-based treatment methods have the advantage that they do not require the introduction of nutrients for microbial growth, do not depend on process control conditions, do not produce biomass accumulation, and therefore do not generate secondary pollution. As shown in Table 1, the biodegradation of BPA by laccase and peroxidase has been extensively researched [27,28].

#### 3.2.1. Biodegradation of BPA by Bacteria

A variety of BPA-degrading bacteria from different environmental substrates have been isolated. The ability of bacteria to degrade BPA in the environment varies with different strains. There were many environmental factors that would affect the degradation efficiency, including biomass, pH value, temperature, BPA concentration, and oxygen.

Among the BPA-degrading bacteria, *Sphingomonas* sp. strains were the most widely used strain, and all isolated *Sphingomonas* sp. strains could use BPA as a sole carbon source. The first BPA-degrading bacteria was isolated from sludge at a sewage treatment plant and identified as the *Sphingomonas* sp. strain MV1 [36,37], which could completely degrade 10 g/L BPA within 4 days. After that, a *Sphingomonas* sp. strain TTNP3 was isolated from the activated sludge samples [38], and it could also degrade BPA [39]. Sasaki et al. isolated *Sphingomonas* sp. strain AO1 with a high degradation rate of BPA from the soil of vegetable planting areas in Japan, which could not only grow in the basal salt mineral (BSM) medium with BPA as a sole carbon source but also completely degrade 115 mg/L of BPA within 6 h (involving the cytochrome P450 system) [40]. Matsumura et al. collected 107 soil samples from various parts of Japan and isolated three strains, namely *Sphingomonas* sp. strains SO11, SO1a, and SO4a. They could grow in the basal salt mineral medium with BPA as a sole carbon source and completely degrade BPA within 48 h [41].

Vijayalakshmi et al. isolated Pseudomonas aeruginosa (PAb1) from the wastewater of the thermal paper industry. PAb1 could use BPA as a sole carbon source to grow on the basal salt mineral medium. Moser, Tesier, and Monad kinetic models were used to record the regression coefficients (R^2^) and semi-saturation coefficients (K_S_) of batch fermentation degradation of BPA, which were 0.94, 0.84, and 0.91, 12.46 g/L, 14.14 g/L, and 9.947 g/L, respectively [42]. In addition, without considering the degradation of BPA, the maximum accurate growth rate of PAb1 was 0.841 h^−1^.

**Figure 4:**
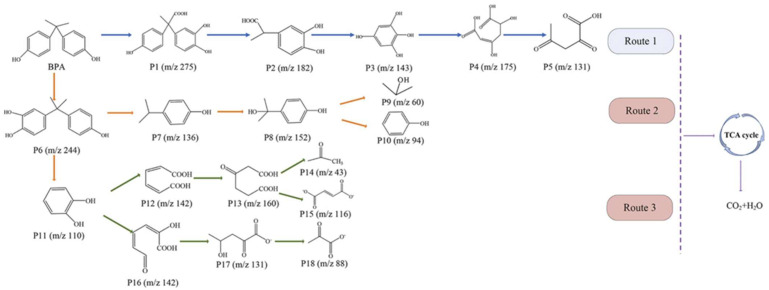
The degradation pathway of BPA by isolated strain SQ-2 [43].

The degradation pathway and molecular mechanism of bacteria in the degradation of BPA have also been explored. Hou et al. proposed that SQ-2 secretes proteins and polysaccharides through Sec-SRP, Tat, and type VI secretion pathways to degrade BPA. The degradation products of BPA then enter the tricarboxylic acid (TCA) cycle and ultimately oxidize to generate carbon dioxide and water [43]. The degradation pathway of BPA by some bacteria is shown in Figure 4.

Numerous bacteria with the capability to degrade bisphenol A (BPA) have been successfully isolated. The majority of these bacteria belong to the Sphingomonas family, including Sphingomonas, Sphingobium, Novosphingobium, and Sphingopyxis [44]. One such strain, MV1, was isolated from the sewage treatment facility of a plastic factory and has demonstrated effective aerobic degradation of BPA and other bisphenol compounds [36]. Moreover, several strains of Pseudomonas also exhibit the ability to degrade BPA. Other bacterial strains, such as Bacillus, Streptomyces, Enterobacterium, and Klebsiella, have been reported to possess the potential for BPA degradation. In the degradation process of BPA, various microbial diversity groups may be involved, primarily through the action of bacteria and fungi. These microorganisms can utilize BPA as a carbon or energy source, breaking it down into simpler organic compounds through metabolic pathways. From Table 2, it is evident that in multiple studies, BPA has been identified as the sole carbon and energy source supporting microbial growth and metabolism, highlighting its favorable bioavailability. Simultaneously, bacteria capable of degrading BPA are widely distributed in various environments, predominantly isolated from environmental media such as soil, sediment, seawater, river water, and even fermented food [40,45]. In addition, due to the toxicity of BPA to bacteria, metabolic limitation, repair cycle, and other factors, the degradation effect of BPA by bacteria was not ideal.

#### 3.2.2. Degradation of BPA by Laccase

Laccases, enzymes found in various organisms, including bacteria, fungi, and plants, possess a wide range of redox potentials. These enzymes play a significant role in the degradation or removal of compounds like BPA [46,47,48]. Bacterial laccases, derived from different bacterial species, exhibit varying redox potentials depending on their specific sources. They are known for their robustness and stability under diverse conditions, making them potentially valuable in BPA degradation processes. Fungal laccases, originating from fungi such as Trametes versicolor and Pleurotus ostreatus, have garnered considerable attention for their ability to oxidize a broad spectrum of substrates. These enzymes often exhibit moderate to high redox potentials, making them effective catalysts for BPA degradation. Plant-derived laccases, while less explored compared to their bacterial and fungal counterparts, also exhibit varying redox potentials. They might offer unique properties and potential applications in the degradation or removal of BPA, although their use in this context might require further investigation [47,48]. Zhao et al. successfully produced recombinant laccase that can efficiently degrade BPA in a medium containing 0.06 IU/mL laccase and 0.5 mmol/L 2,2-Azino-bis (3-ethylbenzothiazoline-6-sulphonic acid) (ABTS) at a temperature of 60 °C and a pH of 4.5. After 60 min of reaction, 95% of the BPA was effectively degraded [29]. Liu et al. used the laccase crude solution prepared by Trametes versicolor and observed even higher efficiency in the degradation of BPA. Under the experimental conditions of 44.6 °C, an initial BPA concentration of 5 mg/L, a pH of 5.2, and a reaction time of 1 h, a degradation efficiency of 97.68% was achieved [30]. Kimura et al. also investigated the experimental parameters of BPA degradation by laccase and found that a temperature of 40 °C and a pH of 7 could achieve complete degradation of BPA. In addition, the researchers discovered that the addition of polyethylene glycol (PEG) could protect the enzyme activity and prevent the capture of laccase molecules in oligomers [31].

Immobilized laccase has emerged as a technology with promising potential in the treatment of BPA residual wastewater. Latif et al. successfully immobilized laccase on copper alginate beads and obtained maximum immobilized laccase activity under the conditions of 3% sodium alginate concentration (*w*/*v*), 0.141 mM CuSO_4_, and 90 min of hardening time. The immobilized laccase displayed notable storage stability and reusability, achieving a degradation efficiency of 96.12% BPA under the conditions of a pH of 5.0, a temperature of 30 °C, 150 rpm, and a reaction time of one hour [32]. Zhang et al. blended functional nano cellulose and sodium alginate to form composite hydrogel beads and then immobilized laccase on them. The degradation rate of BPA was close to 100% after adding ABTS. Due to the low cost of components, composite hydrogel-immobilized laccase held great potential for the degradation of wastewater containing BPA [49]. Wang et al. prepared chitosan-functionalized halloysite nanotubes and immobilized laccase (lac@CS-HNTs) to degrade BPA via an adsorption-covalent binding method. At optimal preparation conditions, the enzyme activity of lac@CS-HNTs was the highest, and the enzyme content reached 60.10 mg/g. Under the conditions of a pH of 5, BPA 40 mg/L, and a temperature of 45 °C, the degradation rate of BPA by lac@CS-NHTs was 87.31% after 12 h of reaction [33]. Additionally, laccase immobilized on monoaminoethyl-N-aminoethyl (MANAE) agarose gel with a load of 12 U/g was able to degrade 100 mg/L BPA with a degradation efficiency of more than 90% within one hour. After 15 cycles of reuse, the immobilized enzyme retained 90% of its BPA degradation ability [50].

In comparison to immobilized laccase, the advantages of free laccase lie in the absence of a complex immobilization process, a relatively straightforward operation, and the ability to maintain high activity during reactions, facilitating the rapid degradation of BPA. However, free laccase may be influenced by environmental conditions during the reaction, making it susceptible to deactivation due to changes in temperature, pH, and other variables. Furthermore, the reusability of free laccase is often limited [47,51]. On the contrary, immobilized laccase, when affixed to a carrier, tends to be more stable and tolerant to variations in environmental conditions. It is relatively easy to separate and recover, enabling reusability and contributing to cost-effectiveness. Nevertheless, immobilized laccase does have limitations, notably the complexity of the immobilization process, which requires certain technical expertise and equipment support [48]. Additionally, there are instances where the immobilization process may impact the activity of laccase, leading to a decrease in catalytic efficiency [52]. Based on the reviewed literature, there is potential to use advanced, environmentally friendly, and cost-effective support materials to immobilize laccase. By doing so, it may be possible to prevent laccase leakage while maximizing its initial activity. Therefore, in the future, various methods could be employed to immobilize laccase on different materials, thus enhancing its stability, efficiency, and recoverability [53].

#### 3.2.3. Degradation of BPA by Peroxidase

Peroxidase and laccase are the most commonly used extracellular enzymes to degrade refractory pollutants by generating highly active and non-specific free radicals. These enzymes possess high oxidation capacity and can effectively degrade BPA. Thus, there are currently two predominant enzymes that catalyze the degradation of phenolic compounds, namely laccase and peroxidase. In the presence of hydrogen peroxide (H_2_O_2_), peroxidase can oxidize phenolic compounds to form phenoxy radicals. Nevertheless, the polymerization reaction between phenoxy radicals can produce polymer precipitation, which can be easily removed from water [54].

Wang et al. developed a two-phase extraction system to extract peroxidase from potato starch processing wastewater and then recovered potato peroxidase solid enzyme powder through dialysis and freeze-drying. The solid enzyme powder could efficiently catalyze the degradation of BPA by hydrogen peroxide. Specifically, after only 10 min of reaction at a pH 6 and a temperature of 23 °C, the degradation rate of BPA reached 99% [34]. Moreover, Taboada-Puig et al. utilized the response surface methodology to degrade BPA by blending versatile peroxidase (VP), sodium malonate (29–43 mM), and MnS0_4_(0.8–1 mM). Based on the results from the response surface methodology, it was found that the highest BPA degradation rate was achieved when the sodium malonate and MnSO_4_ concentrations were at 100 U/L and 29 mM, respectively [55]. To maintain the activity of peroxidase during BPA degradation, a continuous supply of hydrogen peroxide is required.

However, hydrogen peroxide is unstable, making it challenging to store and transport. To address this issue, Tang et al. employed a stainless steel and hemoglobin membrane as anodes and cathodes, respectively, in a membrane-free electrochemical reactor to generate H_2_O_2_ on the cathode in situ. This approach effectively sustained peroxidase activity for continuous BPA degradation [35]. Furthermore, other researchers have immobilized the enzyme in the substrate molecule to prevent enzyme loss during wastewater treatment [56,57]. Current research shows that coupling hydrogen peroxide and peroxidase is a highly effective method for BPA degradation.

Peroxidases and laccases both offer distinct advantages and limitations in BPA degradation. Peroxidases showcase high catalytic efficiency, broad substrate adaptability, and specificity for certain substrates. However, the reliance of peroxidases on auxiliary substances, such as H_2_O_2_, to serve as an oxygen source may elevate operational costs. And the reliance on auxiliary substances might impact the environment due to their production or usage in large quantities. In contrast, laccases demonstrate versatility by functioning across a wide pH range, operating effectively under mild conditions, and having an inherent oxygen source. Nevertheless, laccases may exhibit lower substrate selectivity, necessitating specific engineering or modifications to enhance degradation efficiency for target substrates. The choice between them depends on factors such as the specific environmental conditions, processing requirements, cost considerations, and the desired substrate specificity for a particular application.

### 3.3. Degradation of BPA by Chemical Oxidation

Chemical methods primarily utilize oxidation, where oxidants are used to break down bisphenol compounds and convert them into non-toxic or less toxic substances. Chemical oxidative degradation is a conventional technology for breaking down organic matter. Common chemical oxidants used include potassium permanganate, ozone, and hydrogen peroxide. Table 3 lists the redox potentials of some commonly used oxidants. This paper focused on the introduction of several chemical oxidation degradation methods, including manganese dioxide oxidation, ferrate oxidation, photocatalytic oxidation, and advanced oxidation processes.

#### 3.3.1. Oxidative Degradation of BPA by Manganese Dioxide

Over the years, several scholars have conducted extensive research on the manganese dioxide (MnO_2_) oxidative degradation of various bisphenols. For instance, Gao et al. demonstrated that BPA is highly sensitive to MnO_2_, and the degradation kinetics follow a quasi-first-order reaction within one hour (with a more than 95% confidence level). After that, it follows composite reaction kinetics. When MnO_2_ was excessive relative to BPA, the degradation rate of BPA increased with the increase in the concentrations of BPA and MnO_2_ at a certain pH [58]. On the other hand, the degradation rate decreased with an increase in pH value since protons participate in the reaction as H^+^ (as shown in Equation (1)). Therefore, the decrease in pH leads to an increase in protons available for redox reactions, resulting in faster reaction rates [59].

(1) $$1/2{MnO}_{2}\left(s\right)+2H^{+}+e^{-}\to 1/2{Mn}^{2+}\left(aq\right)+H_{2}O$$

#### 3.3.2. Oxidative Degradation of Bisphenol A by Ferrate

Ferrate is a potent oxidant that exhibits strong oxidation activity throughout the pH range. Its redox potential in acid and alkali solutions can reach 0.7–2.2 V [60]. Furthermore, the reduction of ferrate produces Fe^3+^ ions that can flocculate pollutants [61,62]. Ferrate also boasts strong sterilization and disinfection capabilities, where low doses are sufficient to eradicate most chlorine-resistant microorganisms [63]. Additionally, the treatment process does not produce any toxic by-products [61].

**Figure 5:**
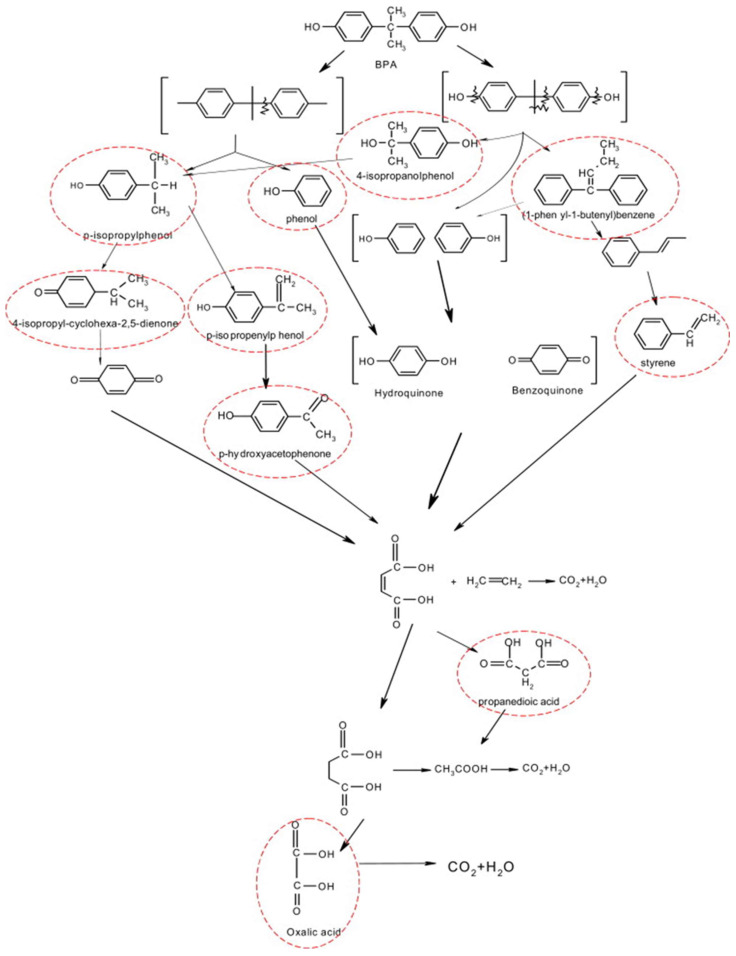
Proposed BPA degradation pathways by ferrate oxidation (main products were circled in red color) [64].

Li et al. conducted a study on the degradation of BPA and steroid estrogens, including estrone, β-estradiol, estriol, and 17α-ethynylestradiol, using potassium ferrate. The study showed that K_2_FeO_4_ has a remarkable degradation efficiency. For instance, when 0.05 mM K_2_FeO_4_ was added to a 0.01 mM BPA solution, BPA underwent bond-breaking reactions and was degraded to *p*-isopropylphenol (IPP), phenol, 4-isopropanolphenol, and (1-phenyl-1-butenyl) benzene, achieving 100% degradation in less than 5 min. Ferrate further degraded the intermediate products to styrene, *p*-hydroxyacetophenone, 4-isopropyl-cyclohexa-2,5-dienone, propanedioic acid, and oxalic acid. Eventually, the BPA was completely mineralized into CO_2_ and H_2_O. Figure 5 depicts the proposed BPA degradation pathways by ferrate oxidation [64].

#### 3.3.3. Photocatalytic Degradation of BPA

Recently, TiO_2_ has been modified through metal/non-metal doping, loading, or composites to enhance electron mobility and improve the photocatalytic degradation of BPA. Chiang et al. compared the photocatalytic activity of TiO_2_ at pHs of 3 and 10, showing that platinum loading of 0.2–1.0 wt% on the TiO_2_ catalyst increased the degradation rate of BPA by 3–6 times compared to the bare TiO_2_ photocatalyst [65]. Kang et al. showed that the use of oxidants (e.g., H_2_O_2_) and iron doping can actively promote BPA degradation on TiO_2_ [66]. Nguyen et al. used 1 wt% ZnFe_2_O_4_ to load TiO_2_, easily achieving five cycles of photodegradation of BPA [67]. Sambaza et al. synthesized a polyaniline anchor 2% Ag@TiO_2_ nanocomposite and successfully degraded 99.8% of BPA under ultraviolet and visible light irradiation, where h^+^ and · O^2−^ played a dominant role [68]. Mei et al. synthesized a TiO_2_/C_3_N_4_ composite photocatalyst (TiO_2_/g-C_3_N_4_) using the hydrothermal method. The results showed that TiO_2_/g-C_3_N_4_ exhibited twice as fast a photodegradation rate of BPA as g-C_3_N_4_ under sunlight, and the composite could completely photodegrade BPA within 20 min [69].

**Figure 6:**
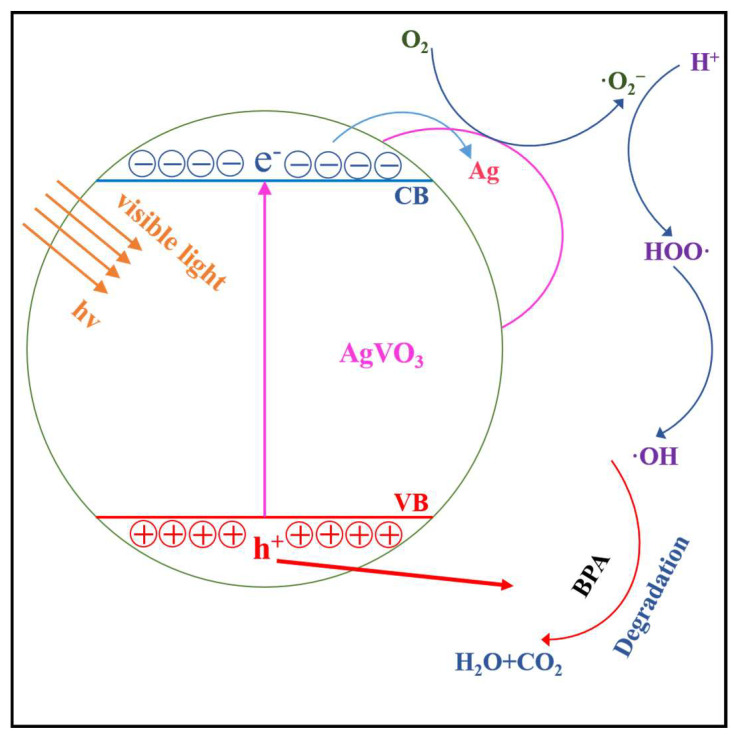
Photocatalytic reaction mechanism of Ag/AgVO_3_ system [73].

In addition to TiO_2_, other materials such as silver, bismuth, and carbon and their modified forms can also be used as photocatalysts to degrade BPA. Ahamad et al. prepared a high-porous photocatalyst, g-C_3_N_4_/MoS_2_-PANI (polyaniline), by in situ polymerization method and found that its degradation effect on BPA was lower than that of the new three-dimensional heterojunction photocatalyst, Fe_3_O_4_/BiOI, prepared by the in situ coprecipitation [70,71]. Chang et al. developed a palladium-modified mesoporous graphite carbon nitride polymer (Pd/mpg-C_3_N_4_) and found that 0.5 g/L Pd/mpg-C_3_N_4_ solids caused complete degradation of BPA [72]. Ju et al. prepared a new type of Ag/AgVO_3_ composite photocatalyst and explored its photodegradation performance on BPA under visible light irradiation. The study showed that adding Ag to β-AgVO_3_ resulted in a synergistic effect, thereby improving β-AgVO_3′_s photocatalytic activity. Ag/AgVO_3_ achieved complete degradation of BPA in 150 min. Finally, the study proposed a photocatalytic degradation mechanism for BPA on the Ag/AgVO_3_ catalyst under visible light irradiation, as shown in Figure 6 [73].

To summarize, Table 4 shows that TiO_2_-based photocatalysts, Ag-based photocatalysts, and other composite photocatalysts demonstrate excellent photocatalytic performance. Photocatalytic technology has great potential for the degradation of BPA. However, there are still some obstacles to improving photocatalyst efficiency. For instance, photocatalyst stability is often a concern in aqueous solution. Additionally, in order to facilitate the recovery and reuse of photocatalyst materials, there needs to be better separation of the photocatalyst from the reactants in future studies.

#### 3.3.4. Advanced Oxidation Degradation of BPA

Over the last three decades, a variety of advanced oxidation technologies (AOTs) have been discovered, rapidly developed, and applied. During AOTs, hydroxyl radicals are generated from UV/H_2_O_2_, UV/O_3_, or photo-Fenton systems, which effectively decompose BPA. Due to their high efficiency, cost-effectiveness, and multifunctionality, AOTs are increasingly used in water treatment plants and other environmental remediation applications. With continued research and development, AOTs are expected to become even more efficient and have broader applications in the future.

**Figure 7:**
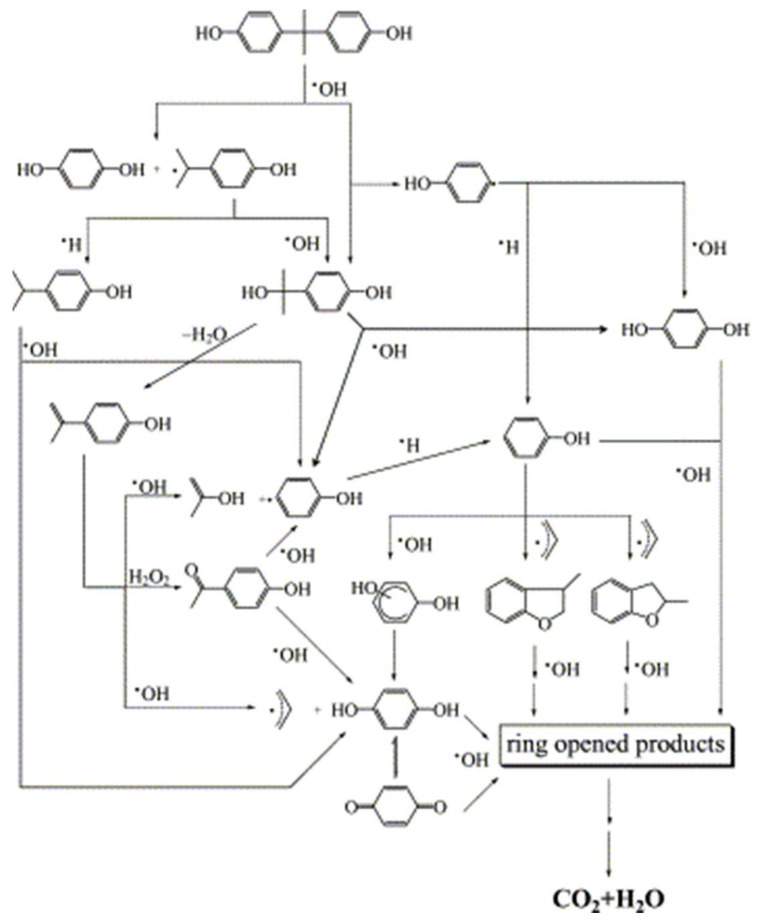
Degradation mechanism of BPA by photo-Fenton process [77].

Zhang et al. conducted a comparative study of the degradation efficiency of BPA by UV, H_2_O_2_, and UV/H_2_O_2_, finding that the UV/H_2_O_2_ process had the best degradation efficiency and reaction time for BPA [74]. Irmak et al. replaced H_2_O_2_ with O_3_ in the UV/H_2_O_2_ process, revealing that BPA can be effectively degraded using O_3_ and UV/O_3_ [75]. Liu et al. conducted a study on the mechanism and optimization methods for the degradation of BPA in aqueous solutions using the combination of ozone, ultraviolet light, and hydrogen peroxide [76]. The study demonstrated that the synergistic effect of ozone, ultraviolet light, and hydrogen peroxide accelerated the degradation of BPA by oxidation and the generation of hydroxyl radicals. Katsumata et al. explored BPA degradation using the photo-Fenton process and found that BPA was completely degraded after 9 min when the optimal ratio of (H_2_O_2_/Fe (II)/BPA) was 9:0.25:1 to 9:0.9:1 [77]. Finally, the study proposed the degradation pathway of BPA, as shown in Figure 7. The photo-Fenton process enhances the oxidation ability of Fenton’s reagent under ultraviolet irradiation, greatly promoting the degradation rate of BPA in the photo-Fenton process.

Overall, these studies suggest that advanced oxidation technologies such as UV/H_2_O_2_, UV/O_3_, and the photo-Fenton process hold great potential for BPA degradation. However, the high operating cost of the photo-Fenton system limits its practical application. Therefore, future research could focus on combining the photo-Fenton method with biochemical methods to fully utilize the advantages of both technologies. This approach could lead to efficient and cost-effective degradation of BPA while minimizing the environmental impact of any by-products generated during the process.

It is important to note that the effectiveness of these oxidation methods can depend on various factors, including the concentration of BPA, the specific oxidizing agent used, reaction conditions (e.g., pH, temperature), and the presence of other substances in the environment. Additionally, the by-products generated during the oxidation process should be carefully assessed to ensure that they are not more toxic than the original compound. Environmental and safety considerations are crucial in selecting and implementing oxidation methods for BPA degradation.

### 3.4. Physical Technology to Remove BPA

Over the past decade, adsorption has emerged as the most widely used method for removing BPA. The raw materials for adsorbents, such as graphene, activated carbon, zeolite, and agricultural waste, are diverse, inexpensive, and easy to maintain. These materials have proven to be promising for large-scale BPA removal applications. The management of adsorbents is one of the key factors in ensuring the efficient operation of the adsorption process. Absolutely, managing adsorbents effectively is crucial throughout the entire process, encompassing selection, storage, regeneration, and treatment. This holistic approach significantly impacts the service life of the adsorbent, cost reduction, and overall efficiency improvement. To successfully utilize adsorption methods for BPA removal, a high level of technical preparation is necessary. This involves ensuring the smooth operation of equipment and instruments and diligent monitoring of various parameters within the adsorption process. Parameters such as adsorbent saturation, temperature, and flow rate, among others, must be continuously assessed to guarantee the efficient removal of BPA. These advancements in adsorption techniques offer significant promise for the efficient and cost-effective removal of BPA. Their successful implementation relies not only on innovation in materials and methods but also on meticulous management and monitoring throughout the entire process.

#### 3.4.1. Adsorption of BPA by Graphene and its Derivatives

Graphene is a two-dimensional crystal composed of a single layer of carbon atoms arranged in a honeycomb lattice structure. It has a very large specific surface area, excellent thermal conductivity, and mechanical properties. According to the Langmuir isotherm, Xu et al. found that at 302.15 K, graphene can adsorb up to 182 mg/g of BPA, particularly under acidic conditions, where it can form strong π−π interactions and hydrogen bonds with BPA [78]. At 298 K, the maximum adsorption capacity of graphene oxide for BPA was found to be 87.80 mg/g, and it reached adsorption equilibrium in 30 min. Moreover, graphene oxide has excellent cyclic adsorption performance and can maintain its adsorption capacity at about 83% after five adsorption cycles [79]. Tang et al. used p-Phenylenediamine-functionalized magnetic graphene oxide nanocomposites (PPD-MGO) to adsorb BPA in an aqueous solution and achieved a maximum adsorption capacity of 155 mg/g at a temperature of 45 °C and a pH of 7. After five adsorption cycles, the adsorption capacity of PPD-MGO remained at 94% [80]. In a different study conducted by Wang et al., it was observed that reduced graphene oxide modified with nitrogen exhibited an enhanced adsorption capacity for BPA compared to reduced graphene oxide without nitrogen. Specifically, the modified material was able to adsorb 1.75 times the amount of BPA in comparison to the unmodified reduced graphene oxide. This suggests that the introduction of nitrogen modifications to the graphene oxide structure can significantly impact its adsorption properties for BPA [81].

#### 3.4.2. Adsorption of BPA by Activated Carbon

Activated carbon is a commonly used adsorption material for the advanced treatment of organic pollutants in water due to its large specific surface area, stable chemical properties, and ease of recycling. Matsushita et al. discovered that when the concentration of BPA was between 0.1 µg/L and 500 mg/L, the adsorption capacity of activated carbon made from kraft paper bags was greater than that of commercial activated carbon. Additionally, at lower concentrations, the rate of BPA adsorption on the activated carbon made from kraft paper bags was faster than that of commercial activated carbon [82].

Gong et al. acidified powdered activated carbon (PAC) with hydrochloric acid and grafted PAC in an aqueous solution with N-isopropylacrylamide (NIPAM) to create PAC-PNIPAM. The maximum adsorption capacity of PAC-PNIPAM was 247.532 mg/g, and the adsorbed PAC could be easily recovered [83].

Liu et al. modified two types of commercial carbon—W20 and W20N—with nitric acid and nitrogen heat treatment, respectively. The findings showed that W20 and W20N had promising adsorption capacities, with the adsorption capacities of BPA reaching 382.12 mg/g and 432.34 mg/g, respectively. However, as the solution pH increased, the adsorption capacity of BPA decreased [84].

In a study by Bautista-Toldo et al., the authors examined the parameters of the BPA adsorption process and discovered that the adsorption of BPA on activated carbon primarily depended on the chemical properties of the carbon surface and the pH value of the solution. The adsorption capacity was reduced due to the hydrophilic properties of carbon and the minerals in the carbon. Electrolytes in the solution facilitated the adsorption process by shielding the positively charged carbon surface and the BPA molecules, thus increasing the adsorbent-adsorbate interactions [85].

#### 3.4.3. Adsorption of BPA by Zeolite

Zeolite is a porous aluminosilicate material with a three-dimensional structure characterized by a permanent negative charge that is balanced by exchangeable cations [86]. The maximum equilibrium adsorption capacity of hydrophobic zeolite for 90 mg/L of BPA was determined to be 125 mg/g at 25 °C [87].

Chen et al. observed that high silica Y-type zeolite can selectively adsorb BPA in landfill leachate with significant concentrations of humic acids, which is possibly due to the hydrophobic interaction between them [88].

Wang et al. modified zeolite with cetyltrimethylammonium bromide to adsorb BPA, resulting in an adsorption capacity ranging from 17.78 to 23.12 mg/g at a pH of 10 [89]. Dong et al. utilized hexadecyltrimethylammonium to modify zeolite synthesized from fly ash (SMZFA) and discovered that the adsorption of BPA by SMZFA was pH-dependent, especially in alkaline conditions [90].

#### 3.4.4. Adsorption of BPA by Agricultural Wastes

The production and processing of agricultural wastes have resulted in their becoming an economically viable, renewable, and eco-friendly adsorption material. Consequently, these agricultural wastes have been used in numerous studies to adsorb BPA, which helps to minimize the release of natural and micropollutants.

For example, in a study conducted by Chang et al., activated carbon derived from rice straw was utilized to adsorb BPA, demonstrating a noteworthy adsorption capacity of 181.19 mg/g [91]. Additionally, Katibi et al. synthesized a novel magnetic biochar derived from palm kernel shells for BPA removal, achieving a maximum removal rate of 94.2%. Moreover, the primary mechanisms governing BPA’s adsorption on the magnetic biochar were identified as π-π electron acceptor-donor interaction, hydrophobic interaction, and hydrogen bonding [92].

Activated carbons prepared from argan nut shell by phosphoric acid also show great potential in BPA adsorption (93%) [93]. As demonstrated by Uzosike et al., they found the adsorption capacity of BPA on activated carbon derived from walnut shell and magnetic activated carbon derived from a walnut shell to be 115.85 mg/g and 166.67 mg/g, respectively [94]. Likewise, in a study by Wang and Zhang et al., magnetic biochar was prepared with excellent reusability [95]. Hayoun et al. discovered that the adsorption performance of sunflower seed shells towards BPA could reach 87.81% after treatment with sulfuric acid [96]. These findings collectively highlight how agricultural waste can be repurposed as an effective and sustainable method for removing BPA from the environment.

#### 3.4.5. Membrane Adsorption of BPA

Membrane technology has been explored as an alternative to traditional treatment processes. Membrane adsorption to remove BPA has several advantages over other methods, including the production of no byproducts or metabolites during operation, which helps to prevent secondary pollution. However, the performance and sustainability of adsorption membranes are influenced by various factors, such as membrane selection, operating conditions, solute concentration, membrane regeneration and maintenance, etc. Therefore, in the future, this technology needs to be optimized by carefully selecting membrane materials, optimizing operating conditions, and conducting monitoring and maintenance work. At the same time, economic costs also need to be weighed against environmental governance benefits to ensure the sustainability of technology.

In a study conducted by Wu et al., the adsorption performance of BPA on polysulfone membranes was investigated. The adsorption of BPA is determined by the properties of polymeric membrane materials, membrane hydrophobicity, and membrane surface roughness. The adsorption rate of BPA by the ultrafiltration membrane in the feed solution was determined to be 32.82% based on mass balance calculations at the conclusion of the filtration process [97].

Wang et al., on the other hand, prepared a multi-walled carbon nanotubes-polysulfone (MWCNTs-PSF) composite membrane with a high hybrid content of carbon nanotubes. The composite membrane significantly improved the adsorption capacity of BPA in an aqueous solution [98].

In another study, Muhamad et al. investigated the BPA removal performance of polyethersulfone (PES) membranes with different silicon dioxide (SiO_2_) nanoparticle contents prepared by a dry-jet wet spinning method. It was found that the PES membrane doped with 2 wt% SiO_2_ had the highest removal rate of BPA (88%) [99]. Overall, these studies suggest that membrane technology can be an effective and sustainable way to remove BPA from the environment.

#### 3.4.6. Adsorption of BPA by Other Adsorbents

In the context of the adsorption process for BPA removal, various materials beyond conventional chemical adsorbents, such as graphene, black phosphorus nanosheets, and activated carbon, have been investigated. For instance, nitrogen-doped carbon nanotubes have exhibited notable efficiency in adsorbing BPA [100]. Silica gel, characterized by its porous structure and extensive surface area, demonstrates a discernible adsorption capacity for BPA. Aluminum oxide, a widely used adsorbent, finds application in removing organic substances, including BPA, from water. Furthermore, certain nanomaterials, like zinc oxide nanoparticles and titanium dioxide nanoparticles, are under consideration for BPA adsorption owing to their distinctive physical and chemical properties.

## 4. Conclusions

Bisphenol compounds have found extensive applications in both industrial processes and daily life, and their significance is anticipated to persist in the decades to come. Consequently, exploring the degradation pathways and mechanisms of these compounds holds paramount importance. This paper provides a comprehensive review of various methods for the degradation of BPA. Photodegradation, identified as a safe and environmentally friendly method, presents significant practical application prospects. The role of photocatalysts is pivotal in this process; thus, the development of efficient photocatalysts remains a key focus for future research. Improving reactor designs is essential to enhancing the performance of photocatalysts, ensuring increased overall adsorption of pollutants on their surfaces, particularly in large-scale operations. Crucially, the degradation products resulting from photocatalysis should not pose a greater threat than the parent pollutants. Moreover, the integration of photocatalytic technology with other synergistic processes, such as sonochemistry and/or biotechnology, should be considered to enhance degradation efficiency. The combination of various methods, leveraging the advantages of biological, chemical, physical, and other approaches, is imperative for the development of efficient and economically viable BPA degradation technologies that can meet diverse treatment requirements.

## Figures and Tables

**Table 1:** Biodegradation of BPA by laccase and peroxidase.

Enzyme	Source	Temperature (°C)	BPA Degradation Efficiency (%)	Reference
Laccase	fungus	60	95	[29]
Laccase	plant	44.6	97.68	[30]
Laccase	bacteria	40	100	[31]
Laccase	plant	30	96.12	[32]
Laccase	plant	45	87.31	[33]
potato peroxidase	plant	23	99	[34]
Peroxidase	bacteria	25	50.7	[35]

**Table 2:** Bacterial isolates capable of degrading BPA.

Strain	Specific Name	Metabolic Type	Source
Novosphingobium	strain TYA-1	the only carbon source	Phragmites
Sphingomonas	strain MV1	the only carbon source	waste water
Sphingomonas	strains AO1, SO11, SO1a, SO4a	the only carbon source	soil
Sphingomonas	Strain BP-7	the only carbon source	seawater
Sphingobium	yanoikuyae strain BP-11R	the only carbon source	river water

**Table 3:** Standard oxidation-reduction potential of several common oxidants.

Oxidant	Standard Redox Potential (V)
Oxygen (O_2_)	0.40
Hydroxyl radical (HO•)	2.80
Atomic oxygen (O•)	2.42
Ozone (O_3_)	2.07
Potassium permanganate (KMnO_4_)	1.51
Chlorine (Cl_2_)	1.36
Hydrogen peroxide (H_2_O_2_)	0.87

**Table 4:** Degradation rate of bisphenol A by various photocatalysts.

Photocatalyst Type	Degradation Rate	BPA Concentration	References
ZnFe_2_O_4_-TiO_2_	98.7%	10 mg/L	[67]
Ag@TiO_2_-PANI	99.8%	5 mg/L	[68]
TiO_2_/g-C_3_N_4_	100%	20 mg/L	[69]
g-C_3_N_4_/MoS_2_-PANI	92.66%	20, 30, 40, 50 and 60 mg/L	[70]
Fe_3_O_4_/BiOI	100%	20 mg/L	[71]
Pd/mpg-C_3_N_4_	100%	20 mg/L	[72]
Ag/AgVO_3_	100%	50 mg/L	[73]

## Data Availability

The authors declare that all data generated or analyzed during this study are included in the published article.
